# Doblin: inferring dominant clonal lineages from high-resolution DNA barcoding time series

**DOI:** 10.1093/bioinformatics/btaf555

**Published:** 2025-10-06

**Authors:** Melis Gencel, David Gagné-Leroux, Adrian W R Serohijos

**Affiliations:** Department of Biochemistry, Université de Montréal, Montréal, QC H3T 1J4, Canada; Robert-Cedergren Center for Bioinformatics and Genomics, Université de Montréal, Montréal, QC H3T 1J4, Canada; Department of Biochemistry, Université de Montréal, Montréal, QC H3T 1J4, Canada; Robert-Cedergren Center for Bioinformatics and Genomics, Université de Montréal, Montréal, QC H3T 1J4, Canada; Department of Biochemistry, Université de Montréal, Montréal, QC H3T 1J4, Canada; Robert-Cedergren Center for Bioinformatics and Genomics, Université de Montréal, Montréal, QC H3T 1J4, Canada

## Abstract

**Motivation:**

The lineage dynamics and history of cells in a population reflect the interplay of evolutionary forces they experience, including mutation, drift, and selection. When the population is polyclonal, lineage dynamics also manifest the extent of clonal competition among co-existing mutational variants. If the population exists in a community of other species, the lineage dynamics could also reflect the population’s ecological interaction with the rest of the community. Recent advances in high-resolution lineage tracking via DNA barcoding, coupled with next-generation sequencing of bacteria, yeast, and mammalian cells, allow for precise quantification of clonal dynamics in these organisms.

**Results:**

In this work, we introduce *Doblin*, an R suite for identifying *do*minant *b*arcode *lin*eages based on high-resolution lineage tracking data. We first benchmarked *Doblin’s* accuracy using lineage data from evolutionary simulations, showing that it recovers the clones’ identity and relative fitness in the simulation. Next, we applied *Doblin* to analyze clonal dynamics in laboratory evolutions of *Escherichia coli* populations undergoing antibiotic treatment and in colonization experiments of the gut microbial community. *Doblin’s* versatility allows it to be applied to lineage time-series data across different experimental setups.

**Availability and implementation:**

*Doblin* is available on CRAN (https://CRAN.R-project.org/package=doblin) and Github (https://github.com/dagagf/doblin).

## 1 Introduction

Microbial populations in natural environments or the laboratory often exhibit polyclonality, characterized by genetic differences among individual cells (Shapiro *et al.* 2012). This polyclonality is due to their high population size and high rates of new genomic variation, including mutations, recombination, or horizontal gene transfer (Eyler 2013, Ackermann 2015). This genomic heterogeneity contributes to phenotypic diversity, such as variable levels of antibiotic tolerance (Balaban *et al.* 2004, Lewis 2007, Shan *et al.* 2017), nutrient uptake (Nikolic *et al.* 2017, Vilhena *et al.* 2018), or growth rates (Kiviet *et al.* 2014, Thomas *et al.* 2018). Within populations comprised of a single species, polyclonality leads to clonal interference, whereby multiple co-existing clones with beneficial mutations, compete for dominance. This competition may result in the interference or inhibition of the spread of one clone by another, thereby influencing the population’s evolutionary trajectory and dynamics (Desai *et al.* 2013). In multiple-species populations, diversity within each species can lead to heterogeneous ecological (inter-species) interactions, with one sub-population of the species competing but another sub-population cooperating with other species in the community (Carrara *et al.* 2015, Ferreiro *et al.* 2018, Hromada *et al.* 2021, Wang *et al.* 2021, Stump *et al.* 2022). Altogether, accurately quantifying the heterogeneity of communities is crucial for understanding evolutionary and ecological dynamics across diverse scientific domains, including microbial evolution (Levy *et al.* 2015, Good *et al.* 2017), microbial engineering (Lopez-Garcia *et al.* 2010, Rompolas *et al.* 2016), and cancer treatment (Landau *et al.* 2013, Mroz *et al.* 2015).

Recent advancements in chromosomal DNA barcoding technology (Blundell and Levy 2014), where a unique DNA barcode introduced into the chromosome is transmitted from parent to daughter cells, have enabled high-resolution measurement of clonal lineages in various organisms, including yeast (Blundell and Levy 2014, Cvijovic *et al.* 2018, Blundell *et al.* 2019), bacteria (Abel *et al.* 2015, Jasinska *et al.* 2020, Vasquez *et al.* 2021, Theodosiou *et al.* 2023, Gencel *et al.* 2025), and mammalian cells (Rogers *et al.* 2018, Ishiguro *et al.* 2025). In bacteria, these studies led to quantitative analysis of the emergence of antimicrobial resistance, while in human cell lines, they facilitated the quantification of cancer recurrence and metastasis (Bhang *et al.* 2015, Nguyen *et al.* 2015, Hata *et al.* 2016, Shaffer *et al.* 2018, Ben-David *et al.* 2018). High-resolution lineage tracking of microbial populations also revealed the coexistence of sub-populations exhibiting unique functional and phenotypic characteristics. Diverse fitness levels and growth rates have been observed, potentially influencing cell fate and differentiation into specific lineages (Lu *et al.* 2011, Pei *et al.* 2017, Biddy *et al.* 2018, Hollmann *et al.* 2020). Furthermore, lineage tracking has shown that evolutionary events, such as the acquisition of beneficial mutations by a sub-population, can lead to an increase in its frequency and significantly impact the collective behavior of the population and its response to environmental changes (Barrick *et al.* 2009, Levy *et al.* 2015, Venkataram *et al.* 2016, Blundell *et al.* 2019, Jasinska *et al.* 2020).

A crucial aspect of analyzing the high-resolution dynamics resulting from DNA barcoding is determining the clonal lineages or sub-populations with similar lineage behaviors. Similarity in DNA barcode dynamics indicate similar fitness among genomes with these barcodes (Blundell and Levy 2014). However, currently, no computational tools are available to identify dominant barcode lineages from high-resolution lineage tracking data. Existing tools leverage single nucleotide variant data from whole-genome sequencing (WGS) to track and reconstruct sub-population clonal dynamics within tumors (Fischer *et al.* 2014, Rubanova *et al.* 2020) and not immediately applicable to DNA barcoding data. While tools like *FitSeq* and *FitSeq2* (Li *et al.* 2018, Li *et al.* 2025) estimate fitness values of DNA barcodes, they do not necessarily identify clonal lineages.

To address this gap, we developed *Doblin*, an R suite for quantifying dominant clonal lineages from DNA barcoding time-series data. This package performs clustering of barcode lineage time-series, based on the idea that similarities in lineage trajectories reflect comparable relative fitness values. Our approach also identifies persistent clonal lineages in the population. As demonstration, we first applied *Doblin* on data of lineage dynamics from forward evolution simulations, showing its ability to identify lineages based on their fitness values. Then, we applied *Doblin* to high-resolution lineage tracking data from a laboratory evolution of *Escherichia coli* under antibiotic resistance and from *E. coli* invasion of the gut microbiome (Gencel *et al.* 2025). *Doblin* is an open-source R package available at CRAN (https://CRAN.R-project.org/package=doblin) and GitHub (https://github.com/dagagf/doblin).

## 2 Methods

**Figure 1.**
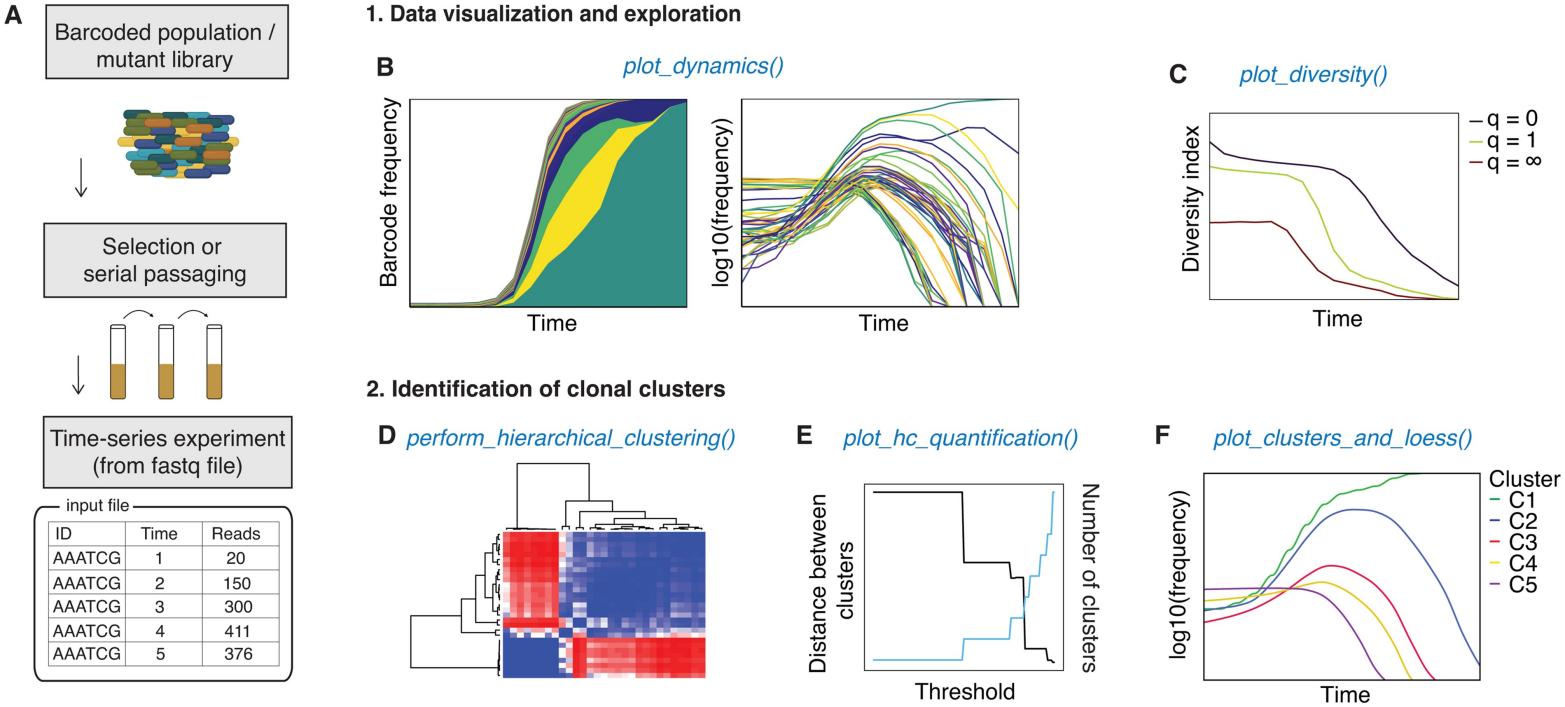
Overview of Doblin. (A) Doblin determines the dominant clonal lineages from high-resolution barcoded population time-series data. The input data can also be time-series of mutants from selection experiments of mutational libraries or variants from CRISPR/Cas screens. The flowchart shows the main functions and steps. (B and C) *Tools for data visualization and overview*. The function *plot_dynamics()* generates linear and logarithmic representations of the barcode lineage frequencies (B). The *plot_diversity()* function quantifies the diversity of chromosomal barcodes using the effective diversity index,Dq=(∑kfkq)1/(1-q) (C). Diversity calculation is based on the count of unique barcodes (q=0), frequency-weighted barcodes (q=1), and the number of dominant barcodes (q=∞). (D–F) *Tools for identification of dominant clonal lineages*: The function *perform_hierarchical_clustering()* compares pairs of frequency trajectories to create a distance matrix based on either Pearson’s correlation or Dynamic Time Warping (DTW). The resulting matrix is used to hierarchically group frequency trajectories with similar behaviors (D). The function *plot_hc_quantification()* evaluates the clusters produced by hierarchical clustering across a range of thresholds (E). Once the optimal threshold has been selected by the user, the function *plot_clusters_and_loess()* computes the LOESS average for each cluster at that hierarchical level. Together, the LOESS averages, or “clonal clusters,” reflect the predominant patterns within the dataset (F). Clonal clusters are ranked according to their final abundance levels.

*Doblin’s* workflow is shown in Fig. 1. It takes as input high-resolution lineage tracking data (Fig. 1A) obtained from DNA barcodes of a microbial population. In principle, any time-series data from pooled competition experiments of variants, such as mutational libraries from deep mutational scans (Fowler *et al.* 2010, Gray *et al.* 2018) or CRISPR screens (Zalatan *et al.* 2015, Ronda *et al.* 2016, Katoh *et al.* 2017), could also be used as input. The input data must be formatted into a table containing barcode identifiers (IDs), timestamps, and read counts associated with each barcode. This data setup is particularly relevant for studies involving serial passaging techniques, where the evolution and propagation of microbes are observed over successive generations.

The first part of Doblin is data visualization and exploration (Fig. 1B and C). Input data dynamics are visualized using the function *plot_dynamics()*, which allows users to choose between logarithmic and linear scales when plotting barcode frequencies (Fig. 1B). The logarithmic scale emphasizes low frequency but persistent cells, while the linear scale highlights dominant clones that have expanded in the population. Since barcode diversity reflects the evolutionary or ecological pressures acting on the population, we also included in Doblin the function *plot_diversity()* (Fig. 1C) to show various diversity metrics used in ecology and evolution. This function calculates the effective diversity index D(t)q=(∑kfkq(t))11-q (Jost 2006, Tuomisto 2010), where $f_{k}^{\;}(t)$ is the frequency of each barcode k and q is the diversity order. When q=0, the index reflects species richness; q=1 gives the exponential of Shannon diversity; and q=∞ reflects dominance by the most frequent barcode.

The second part of Doblin focuses on identifying dominant clonal lineages. This begins by computing a distance matrix that quantifies similarities between barcode frequency trajectories, using either Pearson’s correlation or Dynamic Time Warping (DTW) (Supplementary Information, available as supplementary data at *Bioinformatics* online). After calculating the matrix, users apply *perform_hierarchical_clustering()* to group similar trajectories using Unweighted Pair Group Method with Arithmetic Mean (UPGMA) or Unweighted Pair Group Method using Centroids (UPGMC) via R’s stats::hclust(). Doblin summarizes each cluster with a consensus trajectory generated through locally estimated scatterplot smoothing (LOESS), forming “clonal clusters” that represent dominant population behaviors. It ranks these clusters by their frequency at the final timepoint. To choose the number of clusters, users run *plot_hc_quantification()*, which compares cluster centroids and total cluster counts across cutoff threshold (Supplementary Information, available as supplementary data at *Bioinformatics* online).

## 3 Applications

We used Doblin to analyze dominant clonal lineages in three datasets, each representing a different level of biological complexity: simulations, in vitro evolution, and microbiome colonization. These examples show how Doblin uncovers lineage dynamics from high-resolution barcode data.

### 3.1 Application I—using Doblin to analyze high-resolution lineage data from evolutionary simulations

We first tested Doblin on simulated data where every barcode lineage’s fitness was known. We simulated a population of 10^7^ barcoded cells evolving under a Wright-Fisher model for 1125 generations (Gauthier *et al.* 2019) (Fig. 2, available as supplementary data at *Bioinformatics* online). Because frequency trajectories reflect relative fitness, lineages with similar fitness showed similar shapes. We used Pearson correlation and hierarchical clustering to group these trajectories, identifying six main clusters (C1 to C6 Fig. 2B–D, available as supplementary data at *Bioinformatics* online). C1 contained the dominant lineage that fixed after gaining a beneficial mutation. C2 followed a similar path early on but was later outcompeted. Clusters C3–C6 started with similar frequencies with C1 and C2 but were eventually lost due to lower fitness. At each timepoint, we compared the fitness distributions of clusters (Fig. 2B–D, available as supplementary data at *Bioinformatics* online) and found that Doblin grouped barcodes by their evolutionary outcomes (acquisition of *de novo* mutations followed by fixation or extinction). We also ran Doblin on additional simulations with varying clonal interference and genetic diversity. In all cases, Doblin consistently identified barcode clusters with distinct fitness values (Figs 3–6, available as supplementary data at *Bioinformatics* online).

### 3.2 Application II—using Doblin to extract dominant lineage behaviors from an experimental evolution of *E. coli*

Next, we applied Doblin to high-resolution lineage data from *E. coli* evolved *in vitro* under the antibiotic Trimethoprim (TMP, 0.1 µg/ml, replicate 2) with a population size of ∼3 × 10^7^(Jasinska *et al.* 2020). Compared to the simulated data in Application I, experimental frequency trajectories showed more complex evolutionary dynamics (Figs 7 and 8, available as supplementary data at *Bioinformatics* online). To avoid capturing barcode dynamics due to experimental noise (Jasinska *et al.* 2020), we focused on lineages with mean frequency above 10^−4^ and persistence across at least 12 of 16 time points (Supplementary Information, available as supplementary data at *Bioinformatics* online). Doblin identified clusters of barcodes with similar dynamics (Fig. 7, available as supplementary data at *Bioinformatics* online), and consequently, these lineages showed extensive clonal interference within the population. Lineages like C4, C5, and C6 initially rose but declined as higher-fitness clusters such as C1 and C2 emerged. C1 likely contained pre-existing beneficial mutations, while C2 and C3 showed later, fluctuating increases driven by *de novo* mutations. We also compared the evolutionary dynamics under a lower antibiotic concentration of 0.01 µg/ml TMP (Fig. 8, available as supplementary data at *Bioinformatics* online). Lower TMP delayed the diversity collapse and extended clonal interference, as expected under this scenario of weaker selection pressure. Figures 7 and 8, available as supplementary data at *Bioinformatics* online illustrate the barcode trajectories, cluster structures, and differences in dynamics across TMP levels.

### 3.3 Application III—using Doblin to extract dominant behaviors from abundance time series of *E. coli* invading the microbiome of mouse gut

Last, we used Doblin to analyze high-resolution lineage data from a two-week *E. coli* evolution experiment in the mouse gut, investigating how population heterogeneity influences clonal dynamics in a complex environment (Gencel *et al.* 2025) (Fig. 10, available as supplementary data at *Bioinformatics* online). Our analysis showed that the first clones to colonize the gut did not always become dominant by the experiment’s end (Fig. 10C, available as supplementary data at *Bioinformatics* online). Instead, many early-arriving lineages either disappeared entirely or remained at low frequency (Fig. 10B and C, available as supplementary data at *Bioinformatics* online). Doblin highlighted one clonal cluster, C1, that outcompeted others, pointing to strong adaptation. Other groups, like clusters C4 through C8, stayed at lower levels and showed frequent ups and downs. These patterns might reflect ecological interactions with other gut microbes or the effect of new mutations emerging during colonization.

## 4 Computational time

*Doblin* has very fast runtime but could vary with input size and complexity. To assess this metric, we benchmarked key pipeline steps (*filterData, perform_hierarchical_clustering, filterHC, plotHCQuantification*, and *plot_clusters_and_loess*) using experimental and simulated datasets described in this paper on both Windows (Intel i7-1255U, 16 GB RAM) and Linux (Intel Xeon E5-2643 v3) systems. For the simulated dataset (306336 barcodes, 17 timepoints, 35 MB), these steps took ∼40 CPU seconds on Windows and were twice as fast on Linux. For the experimental dataset (511280 barcodes, 17 timepoints, 60 MB), they were completed in ∼90 CPU seconds on Windows and ∼40 CPU seconds on Linux.

## 5 Discussion

Here, we introduce *Doblin*, a tool designed for the quantitative analysis of polyclonal communities from high-resolution time series data. *Doblin* distinguishes itself by quantifying the fitness of these communities through their evolutionary temporal dynamics, unlike other available tools that primarily rely on fold enrichment for data analysis. To ensure *Doblin’*s accessibility and applicability for a wide range of users, we have provided detailed documentation of its features and utilities.

*Doblin’*s ability to accurately identify clonal clusters relies on the quality of experimental data. Doblin does not automatically correct for experimental DNA barcode artifacts, which could arise from efficiency in genomic extraction or PCR jackpotting. Additionally, our tool primarily focuses on dominant lineages, potentially overlooking low-frequency barcodes that may become extinct early in the experimental timeline. However, users have the flexibility to adjust parameters to include or exclude certain lineages. The quality of the resulting cluster is also expected to improve with better data, such as higher sampling of the DNA barcode time-series.

## Supplementary Material

btaf555_Supplementary_Data

## Data Availability

Data from Application I can be shared upon request to the corresponding author. Data from Applications II and III are available in the National Center for Biotechnology Information Sequence Read Archive under the BioProject accession numbers PRJNA592371 and PRJNA1113343.

## References

[btaf555-B1] Abel S , Abel Zur Wiesch P, Chang H-H et al Sequence tag-based analysis of microbial population dynamics. Nat Methods 2015;12:223–6, 3 p following 226. 25599549 10.1038/nmeth.3253 PMC4344388

[btaf555-B2] Ackermann M. A functional perspective on phenotypic heterogeneity in microorganisms. Nat Rev Microbiol 2015;13:497–508. 26145732 10.1038/nrmicro3491

[btaf555-B3] Balaban NQ , Merrin J, Chait R et al Bacterial persistence as a phenotypic switch. Science 2004;305:1622–5. 15308767 10.1126/science.1099390

[btaf555-B4] Barrick JE , Yu DS, Yoon SH et al Genome evolution and adaptation in a long-term experiment with *Escherichia coli*. Nature 2009;461:1243–7. 19838166 10.1038/nature08480

[btaf555-B5] Ben-David U , Siranosian B, Ha G et al Genetic and transcriptional evolution alters cancer cell line drug response. Nature 2018;560:325–30. 30089904 10.1038/s41586-018-0409-3 PMC6522222

[btaf555-B6] Bhang H-eC , Ruddy DA, Krishnamurthy Radhakrishna V et al Studying clonal dynamics in response to cancer therapy using high-complexity barcoding. Nat Med 2015;21:440–8. 25849130 10.1038/nm.3841

[btaf555-B7] Biddy BA , Kong W, Kamimoto K et al Single-cell mapping of lineage and identity in direct reprogramming. Nature 2018;564:219–24. 30518857 10.1038/s41586-018-0744-4 PMC6635140

[btaf555-B8] Blundell JR , Levy SF. Beyond genome sequencing: lineage tracking with barcodes to study the dynamics of evolution, infection, and cancer. Genomics 2014;104:417–30. 25260907 10.1016/j.ygeno.2014.09.005

[btaf555-B9] Blundell JR , Schwartz K, Francois D et al The dynamics of adaptive genetic diversity during the early stages of clonal evolution. Nat Ecol Evol 2019;3:293–301. 30598529 10.1038/s41559-018-0758-1 PMC6517070

[btaf555-B10] Carrara F , Giometto A, Seymour M et al Inferring species interactions in ecological communities: a comparison of methods at different levels of complexity. Methods Ecol Evol 2015;6:895–906.

[btaf555-B11] Cvijovic I , Nguyen Ba AN, Desai MM. Experimental studies of evolutionary dynamics in microbes. Trends Genet 2018;34:693–703. 30025666 10.1016/j.tig.2018.06.004 PMC6467257

[btaf555-B12] Desai MM , Walczak AM, Fisher DS. Genetic diversity and the structure of genealogies in rapidly adapting populations. Genetics 2013;193:565–85. 23222656 10.1534/genetics.112.147157 PMC3567745

[btaf555-B13] Eyler E. Pouring agar plates and streaking or spreading to isolate individual colonies. Methods Enzymol 2013;533:3–14. 24182913 10.1016/B978-0-12-420067-8.00001-5

[btaf555-B14] Ferreiro A , Crook N, Gasparrini AJ et al Multiscale evolutionary dynamics of host-associated microbiomes. Cell 2018;172:1216–27. 29522743 10.1016/j.cell.2018.02.015 PMC5846202

[btaf555-B15] Fischer A , Vázquez-García I, Illingworth CJR et al High-definition reconstruction of clonal composition in cancer. Cell Rep 2014;7:1740–52. 24882004 10.1016/j.celrep.2014.04.055 PMC4062932

[btaf555-B16] Fowler DM , Araya CL, Fleishman SJ et al High-resolution mapping of protein sequence-function relationships. Nat Methods 2010;7:741–6. 20711194 10.1038/nmeth.1492 PMC2938879

[btaf555-B17] Gauthier L , Di Franco R, Serohijos AWR. SodaPop: a forward simulation suite for the evolutionary dynamics of asexual populations on protein fitness landscapes. Bioinformatics 2019;35:4053–62. 30873519 10.1093/bioinformatics/btz175

[btaf555-B18] Gencel M , Cofino GM, Hui C et al Quantifying the intra- and inter-species community interactions in microbiomes by dynamic covariance mapping. Nat Commun 2025;16:6314. 40628719 10.1038/s41467-025-61368-y PMC12238654

[btaf555-B19] Good BH , McDonald MJ, Barrick JE et al The dynamics of molecular evolution over 60,000 generations. Nature 2017;551:45–50. 29045390 10.1038/nature24287 PMC5788700

[btaf555-B20] Gray VE , Hause RJ, Luebeck J et al Quantitative missense variant effect prediction using large-scale mutagenesis data. Cell Syst 2018;6:116–24 e113. 29226803 10.1016/j.cels.2017.11.003 PMC5799033

[btaf555-B21] Hata AN , Niederst MJ, Archibald HL et al Tumor cells can follow distinct evolutionary paths to become resistant to epidermal growth factor receptor inhibition. Nat Med 2016;22:262–9. 26828195 10.1038/nm.4040 PMC4900892

[btaf555-B22] Hollmann J , Brecht J, Goetzke R et al Genetic barcoding reveals clonal dominance in iPSC-derived mesenchymal stromal cells. Stem Cell Res Ther 2020;11:105. 32138773 10.1186/s13287-020-01619-5 PMC7059393

[btaf555-B23] Hromada S , Qian Y, Jacobson TB et al Negative interactions determine Clostridioides difficile growth in synthetic human gut communities. Mol Syst Biol 2021;17:e10355. 34693621 10.15252/msb.202110355 PMC8543057

[btaf555-B24] Ishiguro S , Ishida K, Sakata RC et al A multi-kingdom genetic barcoding system for precise clone isolation. Nat Biotechnol 2025;1–14. 10.1038/s41587-025-02649-1 PMC13090120 40399693

[btaf555-B25] Jasinska W , Manhart M, Lerner J et al Chromosomal barcoding of *E. coli* populations reveals lineage diversity dynamics at high resolution. Nat Ecol Evol 2020;4:437–52. 32094541 10.1038/s41559-020-1103-z

[btaf555-B26] Jost L. Entropy and diversity. Oikos 2006;113:363–75.

[btaf555-B27] Katoh Y , Michisaka S, Nozaki S et al Practical method for targeted disruption of cilia-related genes by using CRISPR/Cas9-mediated, homology-independent knock-in system. Mol Biol Cell 2017;28:898–906. 28179459 10.1091/mbc.E17-01-0051 PMC5385939

[btaf555-B28] Kiviet DJ , Nghe P, Walker N et al Stochasticity of metabolism and growth at the single-cell level. Nature 2014;514:376–9. 25186725 10.1038/nature13582

[btaf555-B29] Landau DA , Carter SL, Stojanov P et al Evolution and impact of subclonal mutations in chronic lymphocytic leukemia. Cell 2013;152:714–26. 23415222 10.1016/j.cell.2013.01.019 PMC3575604

[btaf555-B30] Levy SF , Blundell JR, Venkataram S et al Quantitative evolutionary dynamics using high-resolution lineage tracking. Nature 2015;519:181–6. 25731169 10.1038/nature14279 PMC4426284

[btaf555-B31] Lewis K. Persister cells, dormancy and infectious disease. Nat Rev Microbiol 2007;5:48–56. 17143318 10.1038/nrmicro1557

[btaf555-B32] Li F , Salit ML, Levy SF. Unbiased fitness estimation of pooled barcode or amplicon sequencing studies. Cell Syst 2018;7:521–5.e4. 30391162 10.1016/j.cels.2018.09.004 PMC6265064

[btaf555-B33] Li F , Tarkington J, Sherlock G. Correction: fit-Seq2. 0: an improved software for high-throughput fitness measurements using pooled competition assays. J Mol Evol 2025;93:183. 39920416 10.1007/s00239-025-10232-0 PMC11850628

[btaf555-B34] Lopez-Garcia C , Klein AM, Simons BD et al Intestinal stem cell replacement follows a pattern of neutral drift. Science 2010;330:822–5. 20929733 10.1126/science.1196236

[btaf555-B35] Lu R , Neff NF, Quake SR et al Tracking single hematopoietic stem cells in vivo using high-throughput sequencing in conjunction with viral genetic barcoding. Nat Biotechnol 2011;29:928–33. 21964413 10.1038/nbt.1977 PMC3196379

[btaf555-B36] Mroz EA , Tward AD, Hammon RJ et al Intra-tumor genetic heterogeneity and mortality in head and neck cancer: analysis of data from the cancer genome atlas. PLoS Med 2015;12:e1001786. 25668320 10.1371/journal.pmed.1001786 PMC4323109

[btaf555-B37] Nguyen LV , Pellacani D, Lefort S et al Barcoding reveals complex clonal dynamics of de novo transformed human mammary cells. Nature 2015;528:267–71. 26633636 10.1038/nature15742

[btaf555-B38] Nikolic N , Schreiber F, Dal Co A et al Cell-to-cell variation and specialization in sugar metabolism in clonal bacterial populations. PLoS Genet 2017;13:e1007122. 29253903 10.1371/journal.pgen.1007122 PMC5773225

[btaf555-B39] Pei W , Feyerabend TB, Rössler J et al Polylox barcoding reveals haematopoietic stem cell fates realized in vivo. Nature 2017;548:456–60. 28813413 10.1038/nature23653 PMC5905670

[btaf555-B40] Rogers ZN , McFarland CD, Winters IP et al Mapping the in vivo fitness landscape of lung adenocarcinoma tumor suppression in mice. Nat Genet 2018;50:483–6. +. 29610476 10.1038/s41588-018-0083-2 PMC6061949

[btaf555-B41] Rompolas P , Mesa KR, Kawaguchi K et al Spatiotemporal coordination of stem cell commitment during epidermal homeostasis. Science 2016;352:1471–4. 27229141 10.1126/science.aaf7012 PMC4958018

[btaf555-B42] Ronda C , Pedersen LE, Sommer MOA et al CRMAGE: CRISPR optimized MAGE recombineering. Sci Rep 2016;6:19452. 26797514 10.1038/srep19452 PMC4726160

[btaf555-B43] Rubanova Y , Shi R, Harrigan CF et al; PCAWG Consortium. Reconstructing evolutionary trajectories of mutation signature activities in cancer using TrackSig. Nat Commun 2020;11:731. 32024834 10.1038/s41467-020-14352-7 PMC7002414

[btaf555-B44] Shaffer SM , Dunagin MC, Torborg SR et al Rare cell variability and drug-induced reprogramming as a mode of cancer drug resistance. Nature 2018; 555:431 -435. 10.1038/nature22794 PMC5542814 28607484

[btaf555-B45] Shan Y , Brown Gandt A, Rowe SE et al ATP-dependent persister formation in *Escherichia coli*. mBio 2017;8. 10.1128/mbio.02267-16 PMC5296605 28174313

[btaf555-B46] Shapiro BJ , Friedman J, Cordero OX et al Population genomics of early events in the ecological differentiation of bacteria. Science 2012;336:48–51. 22491847 10.1126/science.1218198 PMC3337212

[btaf555-B47] Stump SM , Song C, Saavedra S et al Synthesizing the effects of individual-level variation on coexistence. Ecol Monogr 2022;92.

[btaf555-B48] Theodosiou L , Farr AD, Rainey PB. Barcoding populations of pseudomonas fluorescens SBW25. J Mol Evol 2023;91:254–62. 37186220 10.1007/s00239-023-10103-6 PMC10275814

[btaf555-B49] Thomas P , Terradot G, Danos V et al Sources, propagation and consequences of stochasticity in cellular growth. Nat Commun 2018;9:4528. 30375377 10.1038/s41467-018-06912-9 PMC6207721

[btaf555-B50] Tuomisto H. A diversity of beta diversities: straightening up a concept gone awry. Part 1. Defining beta diversity as a function of alpha and gamma diversity. Ecography 2010;33:2–22.

[btaf555-B51] Vasquez KS , Willis L, Cira NJ et al Quantifying rapid bacterial evolution and transmission within the mouse intestine. Cell Host Microbe 2021;29:1454–68.e4. 34473943 10.1016/j.chom.2021.08.003 PMC8445907

[btaf555-B52] Venkataram S , Dunn B, Li Y et al Development of a comprehensive genotype-to-fitness map of adaptation-driving mutations in yeast. Cell 2016;166:1585–96.e22. 27594428 10.1016/j.cell.2016.08.002 PMC5070919

[btaf555-B53] Vilhena C , Kaganovitch E, Shin JY et al A Single-Cell view of the BtsSR/YpdAB pyruvate sensing network in *Escherichia coli* and its biological relevance. J Bacteriol 2018;200. 10.1128/jb.00536-17 PMC5717152 29038258

[btaf555-B54] Wang X , Peron T, Dubbeldam JL et al Interspecific competition shapes the structural stability of mutualistic networks. *CSF* 2023;172:113507.

[btaf555-B55] Zalatan JG , Lee ME, Almeida R et al Engineering complex synthetic transcriptional programs with CRISPR RNA scaffolds. Cell 2015;160:339–50. 25533786 10.1016/j.cell.2014.11.052 PMC4297522

