# Supplementary material for: Doblin: inferring dominant clonal lineages from high-resolution DNA barcoding time series
Source: Bioinformatics. 2025 Oct 6;41(10):btaf555. doi: 10.1093/bioinformatics/btaf555 (PMC12553330; doi:10.1093/bioinformatics/btaf555)
Supplement: btaf555_Supplementary_Data [file btaf555_supplementary_data.pdf]

Supplementary Information for

Doblin: Inferring dominant clonal lineages from high-resolution DNA barcoding time series

Melis Gencel[1,2,#], David Gagné-Leroux[1,2,#], and Adrian W.R. Serohijos[1,2,*]

[1]Department of Biochemistry, Université de Montréal, 2900 Édouard-Montpetit, Montréal, Quebec, Canada H3T 1J4, [2]Robert-Cedergren Center for Bioinformatics and Genomics, Université de Montréal, 2900 Édouard-Montpetit, Montréal, Quebec, Canada H3T 1J4

[#]These authors contributed equally.

*Corresponding author. Email: adrian.serohijos@umontreal.ca

Supplementary Method

1. Identification of dominant clonal lineages *3*

1.1. Pairwise distance matrix between DNA barcode lineage time series **3**

1.2. Hierarchical clustering **3**

1.3. Effect of technical noise on clonal cluster stability **4**

2. Detailed description of example applications *7*

2.1. Application I: Using *Doblin* to analyze high-resolution lineage data from evolutionary simulations **7**

2.2.* Application II: Using Doblin to extract dominant lineage behaviors from an experimental evolution o*f E. coli **13**

2.3. Application III: Using *Doblin* to extract dominant behaviors from abundance time series of *E. coli* invading the gut microbiome of mouse **16**

Supplementary Figures

Supplementary Figure 1 Robustness of Doblin clustering under noise. **5**

Supplementary Figure 2 Application 1: Using Doblin to analyse high-resolution lineage data from evolutionary simulations. **8**

Supplementary Figure 3 Application of Doblin to evolutionary simulations with standing genetic variation (mean fitness effect s = 0.1) and *de novo* mutations. **9**

Supplementary Figure 4 Application of Doblin to evolutionary simulations with standing genetic variation (mean fitness effect s = 0.05). **10**

Supplementary Figure 5 Application of Doblin to evolutionary simulations with standing genetic variation (mean fitness effect s = 0.01) and beneficial *de novo* mutations. 11
Supplementary Figure 6 Using Doblin to analyze high-resolution lineage data from evolutionary simulations with DTW distance.. 12
Supplementary Figure 7 Application II: Doblin applied to a month-long experimental evolution of *E. coli* under antibiotics. 14
Supplementary Figure 8 Doblin applied to a month-long experimental evolution of *E. coli* under lower antibiotic regime.. 15
Supplementary Figure 9 Dynamics of barcoded lineages frequencies in simulation and experiments to which Doblin has been applied.. 16
Supplementary Figure 10 Application III: Using Doblin to extract dominant behaviors from abundance time series of *E. coli* invading a mouse gut microbiome........................ 17

1. Identification of dominant clonal lineages

1.1. Pairwise distance matrix between DNA barcode lineage time series

After the data exploration and visualization, the second step in *Doblin* is identifying dominant clonal lineages. These clonal lineages are estimated by clustering barcode time series according to their shape and temporal similarities. To improve the accuracy of the clustering, barcode IDs with insufficient time-points are filtered out. This filtering requires users to specify both the number of time points to be included and a frequency threshold, ensuring that only dominant and persistent lineages are retained for the downstream analysis. These considerations depend on the experimental accuracy and study design (Gencel, et al., 2025; Jasinska, et al., 2020).

Identifying the dominant clonal lineages begins with assessing the similarities between pairs of DNA bacode frequency trajectories. This step involves computing a distance matrix. Two methods are available for computing the matrix: one relies on Pearson's correlation, while the other utilizes Dynamic Time Warping (DTW). Users can choose their preferred method based on experimental data and intended analysis. The primary objective of computing the distance matrix is to hierarchically group frequency trajectories exhibiting similar behaviors. With Pearson's correlation method, the distance ΔF_{xy}between two frequency trajectories f_x and f_y is computed as:

$$\Delta F_{xy} = 1 - \rho\big(log_{10} f_x, log_{10} f_y\big) \tag{1}$$

where $\rho\big(log_{10} f_x\,, log_{10} f_y\big)$ represents the Pearson's correlation coefficient between the trajectories. A distance close to 0 signifies a positive correlation between the lineages, while a distance nearing 2 indicates negative correlation.

Conversely, DTW adopts a different approach for time-series comparison, prioritizing shape, and overall patterns over point-to-point similarities. It quantifies the alignment between two time series by determining the optimal warping path, thereby accommodating local temporal shifts and variations. The computation of DTW involves creating a cumulative distance matrix $D(i, j)$ between the ith element of f_x and jth element of f_y defined as:

$$D(i, j) = d\big(f_x[i], f_y[j]\big) + \min\big(D(i-1, j), D(i, j-1), D(i-1, j-1)\big) \tag{2}$$

Here, $d\big(f_x[i], f_y[j]\big)$ represents the local distance between the ith element of f_x and jth element of f_y, which can be either the Euclidean or Manhattan distance. DTW dynamically computes the cumulative distance matrix and identifies the optimal alignment across all warping paths between the two time-series.

The choice between Pearson's correlation and DTW depends on the specific characteristics of the time-series and the objectives of the analysis. Pearson's correlation is preferred when quantifying the strength and direction of the linear relationship between trajectories. While DTW is particularly well-suited for capturing nuanced relationships in data exhibiting temporal shifts and non-linear patterns, such as stretching or compression. These patterns are commonly observed in the context of varying growth rates, transient gut dynamics or ecological interactions.

1.2. Hierarchical clustering

The calculated distance matrix is used to hierarchically group frequency trajectories that exhibit similar behavior, as quantified by Pearson's correlation or Dynamic Time Warping (DTW). This hierarchical clustering is performed by the function *perform_hierarchical_clustering()*, which applies algorithms such as the Unweighted Pair Group Method with Arithmetic Mean (UPGMA) or the Unweighted Pair Group Method with Centroid Averaging (UPGMC), as implemented in R's *stats::hclust()* function (R Core Team, 2013). The resulting clusters group DNA barcode trajectories, which are collectively summarized by a consensus trajectory using locally estimated scatterplot smoothing (LOESS) a non-parametric regression method provided by R's *loess()* function. LOESS fits local polynomial regressions to subsets of the data, applying a default tri-cube weighting function to assign weights based on proximity. These LOESS-derived consensus trajectories, referred to as "clonal clusters," represent the dominant behaviors in the dataset and are ranked according to their frequency at the final timepoint (presumably corresponding to the end of the experiment).

As hierarchical clustering yields a tree structure, determining the number of resulting clonal clusters relies on a cutoff threshold. Traditional clustering evaluation metrics such as the Silhouette Coefficient and Dunn's Index are often used to determine the optimal number of clusters (Raihan, 2023). However, with time series data, these metrics may not adequately assess clustering quality (Dunn, 2008). In Doblin, we determine the optimal number of clusters by comparing the distance between cluster centroids and the resulting cluster counts across various cutoff thresholds. This is done by the function *plot_hc_quantification()* and is guided by several user-defined considerations. First, we improve robustness against sequencing errors by allowing users to filter out clusters containing fewer than a user-specified number of lineages. Clusters of high-frequency barcodes, even if limited in number of member lineages, are retained. Typically, this is the case when only a few barcodes sweep the population, as shown in the Application I below. Secondly, we calculate the distances between clonal clusters by computing the Euclidean distance between their LOESS averages. Our method focuses on identifying the crossover point where the minimum distance between cluster centroids (their LOESS averages) and the overall cluster count converges. Setting the threshold too low may produce many clusters that are similar. Conversely, a high threshold may yield too few clusters, grouping distinct clonal lineage dynamics together. We note that the cluster count and inter-cluster distance curves are not normalized or dimensionally matched. The intersection serves as a visual heuristic, not a formal optimization, to help identify a reasonable balance between trajectory similarity and grouping resolution. Our approach enables the user to explore the data and select an optimal cutoff threshold.

1.3. Effect of technical noise on clonal cluster stability

To examine the impact of technical variability on clonal clustering, we performed controlled down-sampling of simulated barcode dynamics (Supplementary Figure 1A) (Gauthier, et al., 2019). The simulations provided a "ground truth" dataset without noise, and we introduced perturbations by resampling barcode frequencies with a binomial process (Supplementary Figure 1A). At randomly chosen time points, the number of cells contributing to the dataset was reduced by factors ranging from 10^{-1} to 10^{-6}, These reductions mimic sources of variation, including limited DNA yield during extraction, uneven recovery across time points, and differences in sequencing depth during library preparation.

Each perturbed dataset was analyzed with Doblin, and the procedure was repeated 100 independent times to account for stochastic effects (Supplementary Figure 1B–C). We assessed robustness by comparing clusters in downsampled versus original data. Cluster identity was evaluated using Jaccard similarity, where values near 1 indicate strong agreement, and lineage dynamics were compared using an average trajectory distance metric. Across replicates and sampling levels, the dominant cluster (C1) remains stable in both Jaccard similarity and trajectory dynamics in Supplementary Figure 1B–C until input is reduced to ~10^4 cells (10^{-5} down-sampling). In contrast, smaller clusters (C2 and C3) begin to vary once input falls below ~10^5 cells, as indicated by broader error bars and lower Jaccard similarity values. These analyses show that Doblin recovers dominant clusters, while rare lineages are more sensitive to extreme reductions in input (Supplementary Figure. 1C–D).

These results demonstrate that Doblin can reliably identify clonal clusters under different sampling conditions. Only when the effective input dropped below 10^5 cells did we observe degradation in cluster dynamics. Finally, we note that Dynamic Time Warping provides an alternative similarity metric in Doblin and useful in scenarios that involve environmental perturbations of multi-species consortia, where temporal shifts between trajectories are expected. We note that other sources of experimental variability, such as PCR jackpotting, uneven amplification, or stochastic fluctuations in library preparation, have been examined in previous work (Gencel, et al., 2025; Kinsler, et al., 2023) and exert only minor effects when sufficient cell numbers are sequenced. Together, this supports the robustness of our approach to common sources of technical noise.

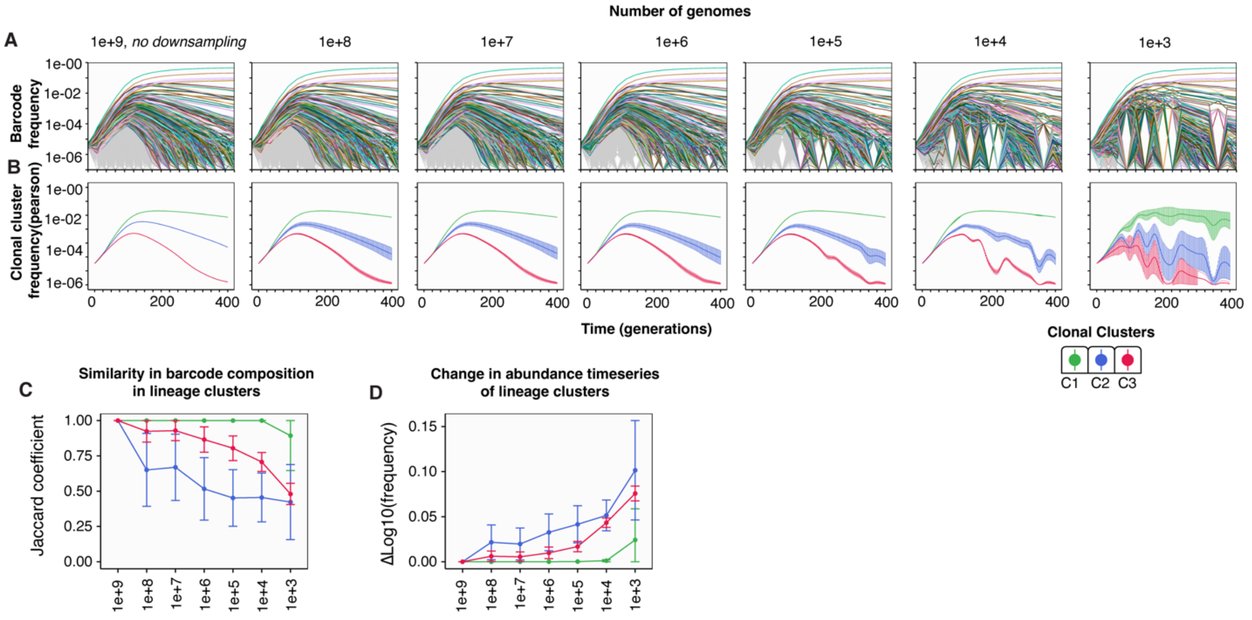

Supplementary Figure 1 Robustness of Doblin clustering under noise.

A, Barcode frequency trajectories generated from Wright–Fisher simulations, with the population size reduced stepwise by factors of ten down to 10^3 cells. **B** Clonal clusters inferred with Doblin using Pearson correlation. Solid lines show the mean trajectories of dominant clusters across 100 independent down-sampling replicates, and error bars indicate variability across replicates. **C–D,** Evaluation of cluster stability between full and down-sampled data. Panel C reports overlaps in

barcode composition, while panel D shows differences in cluster trajectories. Dominant clusters remained stable across conditions, but the integrity of low-frequency clusters deteriorated once the effective number of cells dropped below ~10^5

2. Detailed description of example applications

2.1. Application I: Using *Doblin* to analyze high-resolution lineage data from evolutionary simulations

A barcode's frequency trajectory reflects its fitness relative to the population's mean fitness. Barcodes with similar frequency trajectories typically exhibit comparable fitness levels. Since our approach focuses on clustering lineages based on the shape of their trajectories, the clonal clusters generated by Doblin are expected to represent groups with varying fitness levels. We benchmarked Doblin's accuracy using simulated data with known fitness values to validate its effectiveness in identifying dominant clones with distinct fitness profiles. Specifically, we simulated the evolution of a population of N = 10^7 barcoded cells (with 10^5 unique barcodes) using a Wright–Fisher process with selection (Gauthier, et al., 2019). In this model, generations are non-overlapping, and the population evolves over time through both random sampling (genetic drift) and fitness-based selection. In each generation, cells with higher fitness have a greater probability of producing offspring.

Simulations were performed at the level of individual cells rather than barcodes. This means that cells sharing the same barcode can diverge in fitness over time due to independent mutation events. This design reflects realistic experimental conditions: in typical lineage tracing experiments, such as those described by (Jasinska, et al., 2020), the barcoding process includes a clonal expansion step during which spontaneous mutations can occur, leading to heterogeneity within a single barcode group.

Supplementary Figure 2A illustrates the resulting frequency trajectories over the course of the simulation. Initially, all barcoded cells were assigned identical fitness scores $(f = 1)$, and each cell's frequency was tracked over 1,125 generations. During the simulation, cells underwent *de novo* mutations at a rate of 10^{-5} mutations per genome per replication. The fitness effects of these mutations followed a normal distribution with a mean $\mu = -0.02$ and a standard deviation $\sigma = 0.02$, reflecting a bias toward deleterious effects (85% deleterious, 15% beneficial). Supplementary Figure 8A shows these trajectories in a Muller plot, depicting their variation over time on a linear scale, which highlights the most dominant lineages.

To identify dominant behaviors within the simulated abundance time series, we clustered the frequency trajectories using Pearson's correlation. Only trajectories with an average frequency greater than 10^{-4}, and that persisted for at least 12 of the 46 time points, were retained for clustering. Figure 1D shows the resulting clusters obtained via hierarchical clustering. By selecting a threshold at the intersection of two criteria—the number of clusters and inter-cluster distance (Supplementary Figure 2C),we identified six distinct groupings (C1–C6). Examination of the clusters revealed that lineages with similar trajectory shapes were grouped together (Figure 3D). Cluster 1 (C1, green trajectory) contained the most frequent barcode that ultimately swept through the population. Cluster 2 (C2, blue trajectory), the second most frequent clonal cluster, initially mirrored C1's trajectory but began to diverge around generation 450, at which point C1 acquired a beneficial mutation that allowed it to outcompete C2. Clusters C3 to C6, despite starting with frequencies comparable to C1, experienced extinction at different stages of the simulation.

At selected time points (indicated by arrows in Supplementary Figure 2B), we show the distribution of fitness values for cells within each clonal cluster (Supplementary Figure 2E). At $t = 0$, all clones had identical fitness levels. As the simulation progressed, clonal trajectories diverged due to differences in fitness. Clonal clusters with lower fitness began to go extinct by generation 400. Clusters C1 and C2 showed similar fitness distributions and frequency trajectories until generation 450, when a beneficial mutation in C1 increased its fitness, eventually allowing it to

dominate the population. In contrast, C2 maintained a stable fitness level before a later mutation around generation 750 provided a temporary fitness boost. However, this was insufficient to surpass C1, leading to C2's eventual extinction.

Together, these results demonstrate that Doblin's inferred clonal clusters are distinguishable by their fitness profiles, as expected from a shape-based clustering approach We further applied Doblin to evolutionary simulations with varying levels of polyclonality and clonal interference by altering the degree of standing genetic variation and the effects of *de novo* mutations (Supplementary Figures 3–5). Across these diverse evolutionary scenarios, Doblin consistently identified clonal clusters representing lineages with distinct fitness levels. In addition, we also applied Dynamic Time Warping (DTW) to the simulation in Supplementary Figure 2 (Supplementary Figure 6). Instead of using correlation to define clonal clusters, we used DTW distance to capture temporal similarity in frequency trajectories. We also present the DTW alignment paths for the top three ranked clones in Supplementary Figure 6E-F, ranked according to their average frequencies over time in the simulation.

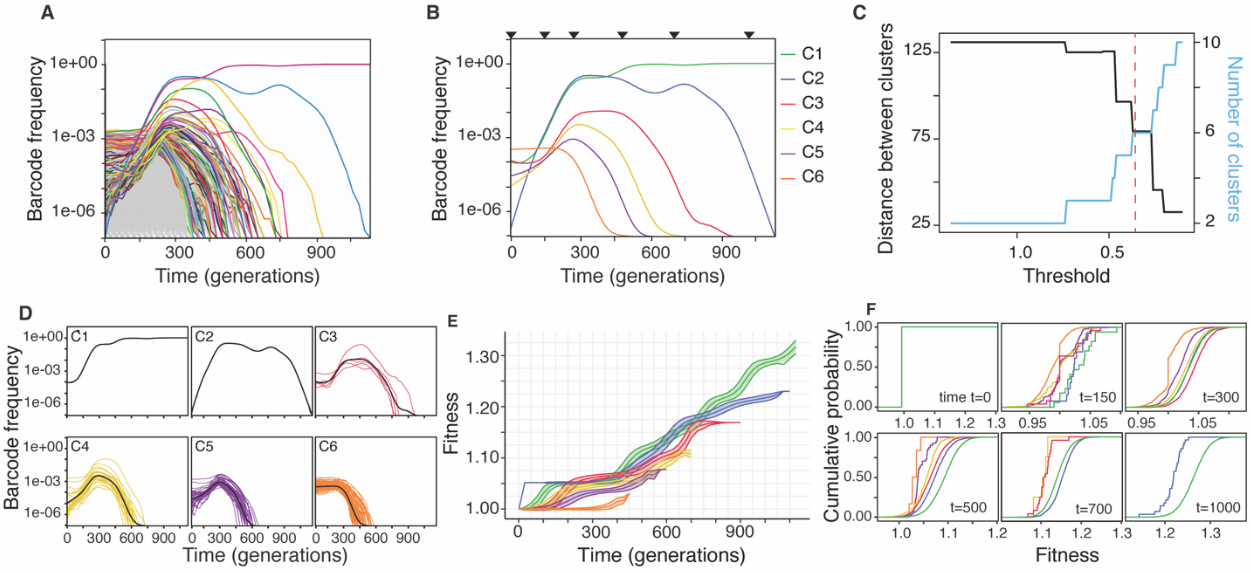

Supplementary Figure 2 Application 1: Using Doblin to analyse high-resolution lineage data from evolutionary simulations.

(**A**) Forward evolutionary simulations of bacterial populations using the Wright-Fisher process implemented in *SodaPop* (Gauthier, et al., 2019). Approximately 10^7 barcoded cells were initially assigned with equal fitness ($f = 1$). During the evolution, cells acquired *de novo* mutations at a rate of 10^{-5} per genome per replication. The selection coefficient s of the mutant is drawn from a normal distribution (mean = -0.02, s.d. = 0.02) and updates the fitness of the mutant as $f_{mutant} = f_{mutant}(1 + s)$. The top 1000 barcodes with a mean frequency over time greater than 10^{-4} are colored uniquely, whereas the rest are shown in gray. (**B**) Consensus trajectories, or centroids, of the 6 clonal clusters identified by *Doblin*. These trajectories correspond to the dominant behaviors of the forward evolutionary simulations. The distribution of fitness across the population for specific time points (arrows) are shown in panel E. (**C**) The quantification of hierarchical clustering shows how the Euclidean distance between consensus trajectories (black curve) varies with the number of subsequent clusters (blue curve). The intersection between these two curves (red dashed line) represents the heuristically optimal clustering cutoff, which generated 6 clonal clusters (i.e.,

C1 – C6). (**D**) Composition of each clonal cluster. These clusters were obtained using a pairwise distance matrix based on Pearson's correlation. Lineages with mean frequency over time greater than 10^{-4} and persisting for at least 12 time points out of 46 are included in the clustering. The consensus trajectory (black curve) for a cluster is obtained by local regression (LOESS). Note, Cluster 1 (C1) contains the barcode lineage that swept the population (see panel A). (**E**) Mean fitness plus standard deviation $(mean \pm s.d/2)$ of clonal clusters. Colors correspond to the fitness of barcodes in a clonal cluster determined by our tool Doblin (Supplementary Figure 2B). Notably, the dominant clone C1 has the highest fitness, the less dominant clones have lower fitness. Additionally, low fitness clone (C6) are the barcodes that become extinct first (see Supplementary Figure 2B), while the most fit clone (C1) is one showing the sweep (see Supplementary Figure 2B). Note that the fitness values end when the clonal cluster becomes extinct.(**F**) Cumulative distribution functions (CDFs) of the fitness of all cells in each clonal cluster at the indicated time point. All cells (and clones) have $f = 1$ at the start of the simulation (all CDFs are overlapping). As the simulation progresses, the fitness distributions diverge, as each clonal cluster acquired different mutations.

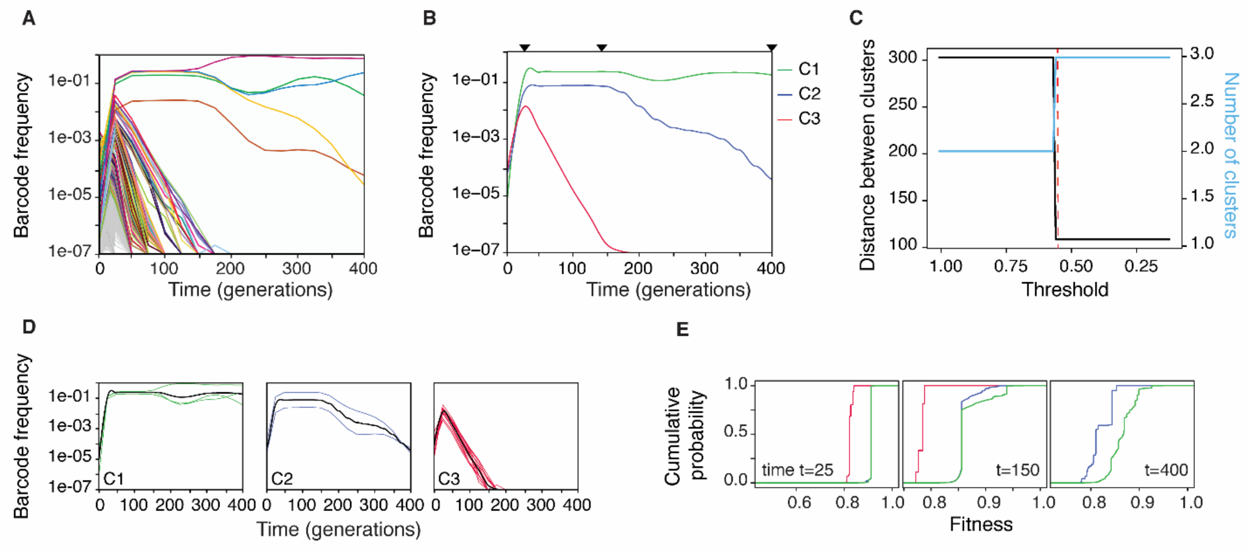

Supplementary Figure 3 Application of Doblin to evolutionary simulations with standing genetic variation (mean fitness effect s = 0.1) and *de novo* mutations.

(**A**) Similar to Supplementary Figure 2, but cells exhibit different fitness levels at $t = 0$ due to pre-existing mutations, with a mean fitness effect of $s = 0.1$. During the evolution, cells acquired *de novo* mutations at a rate of 2.5×10^{-4} per genome per replication. These new mutations have selection coefficients that follow a Gaussian distribution, with 90% of the values being negative (indicating detrimental effects on fitness) and 10% being positive (indicating beneficial effects). The mean selection coefficient for the beneficial mutations is $s = 0.01$. Lineages with a minimum frequency of $5\text{x}10^{-5}$ are colored uniquely, while all other lineages are in gray. (**B**) The 3 clonal clusters identified by *Doblin*. The distribution of fitness across the population for specific time points (arrows) are shown in panel E. (**C**) The quantification of hierarchical clustering shows how the Euclidean distance between clonal clusters (black curve) changes depending on the number of subsequent clusters (blue curve). The intersection between these two curves (red dashed line) represents the heuristically optimal clustering, which generated 3 clonal clusters (i.e., C1 – C3). (**D**) Composition of each clonal cluster. We included lineages in our analysis only if they demonstrated a mean frequency greater than 5×10^{-5} and were present for a minimum of 2 out of 17 time points. (**E**) Cumulative

distribution functions (CDFs) of the fitness of all cells in each clonal cluster at the indicated time point. *Doblin* and the clonal clusters group barcoded cell lineages of similar fitness.

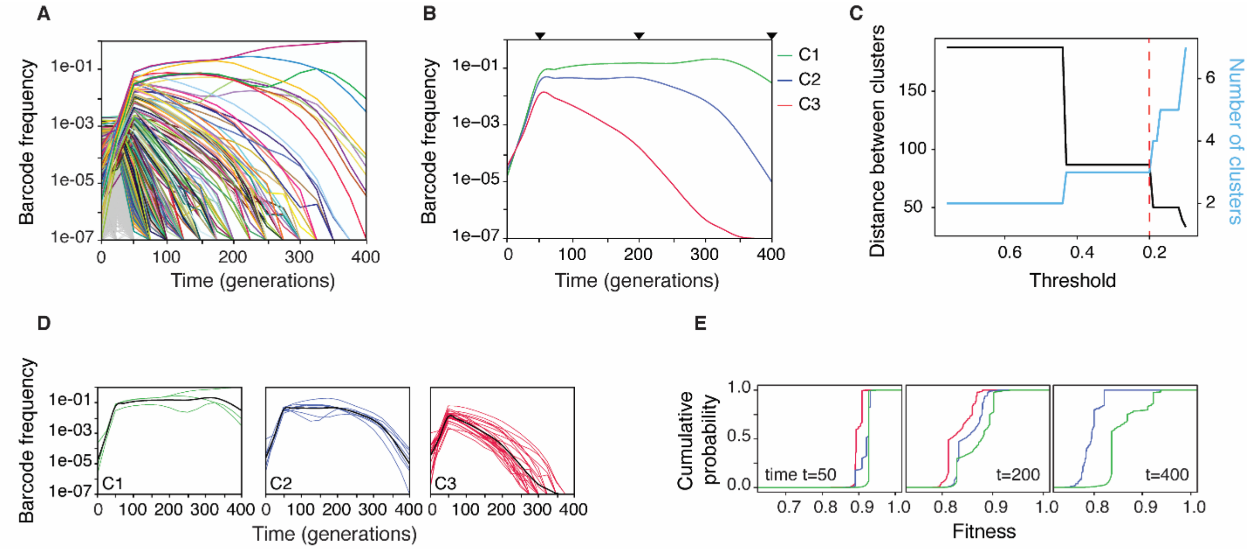

Supplementary Figure 4 Application of Doblin to evolutionary simulations with standing genetic variation (mean fitness effect s = 0.05).

(**A**) Similar to Supplementary Figure 2 but exhibiting pre-existing mutations with a mean fitness effect of $s = 0.05$. (**B**) The 3 clonal clusters identified by *Doblin*. (**C**) Criteria for finding the optimal number of clusters. (**D**) Composition of each clonal cluster. We included lineages in our analysis only if they demonstrated a mean frequency greater than 5×10^{-5} and were present for a minimum of 10 out of 17 time points. (**E**) Evolution of CDFs of the fitness levels for each clonal cluster. Color descriptions for B-E are similar to Supplementary Figure 2-3.

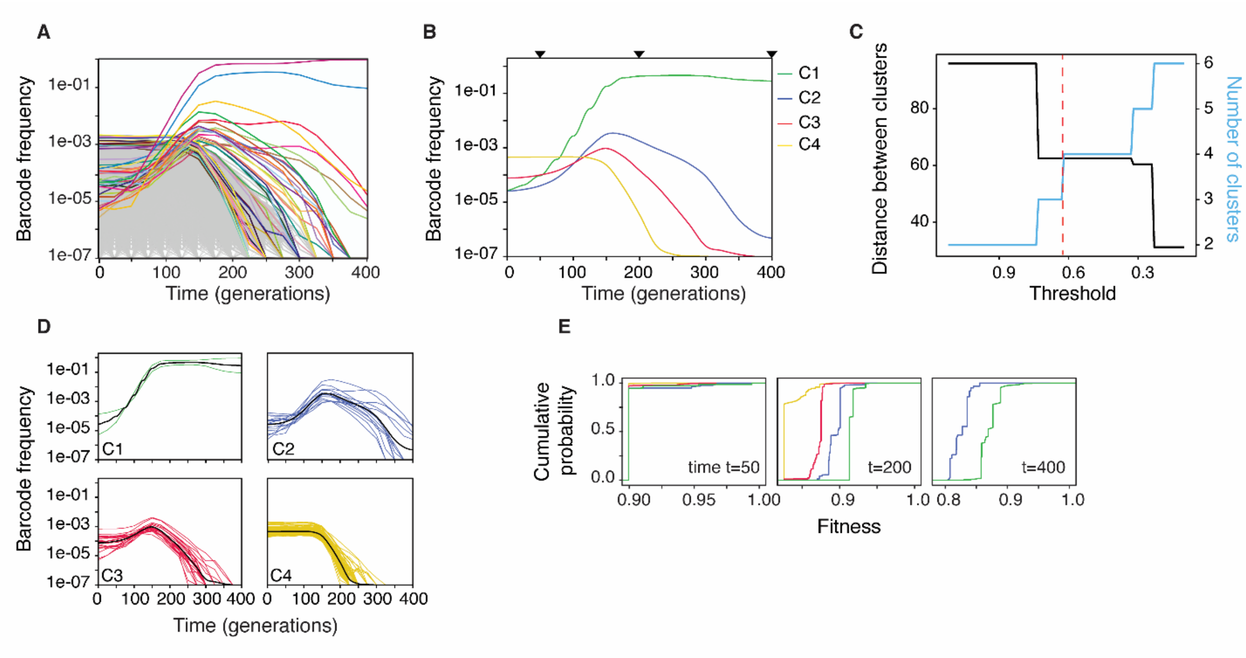

Supplementary Figure 5 Application of Doblin to evolutionary simulations with standing genetic variation (mean fitness effect s = 0.01) and beneficial *de novo* mutations.

(**A**) Similar to Supplementary Figure 2 but exhibiting pre-existing mutations with a mean fitness effect of $s = 0.01$. During the evolution, cells acquired *de novo* mutations at a rate of 2.5×10^{-4} per genome per replication. These new mutations have selection coefficients that follow a Gamma distribution, with 100% of the distribution being positive. The mean selection coefficient for the beneficial mutations is $s = 0.01$. Lineages with a minimum frequency of 10^{-4} are colored uniquely, while all other lineages are in gray. (**B**) Consensus trajectories of the 4 clonal clusters identified by *Doblin*. (**C**) Criteria for finding the optimal number of clusters. (**D**) Composition of each clonal cluster. We included lineages in our analysis only if they demonstrated a mean frequency greater than 5×10^{-5} and were present for a minimum of 8 out of 17 time points. (**E**) Evolution of CDFs of the fitness levels for each clonal cluster.

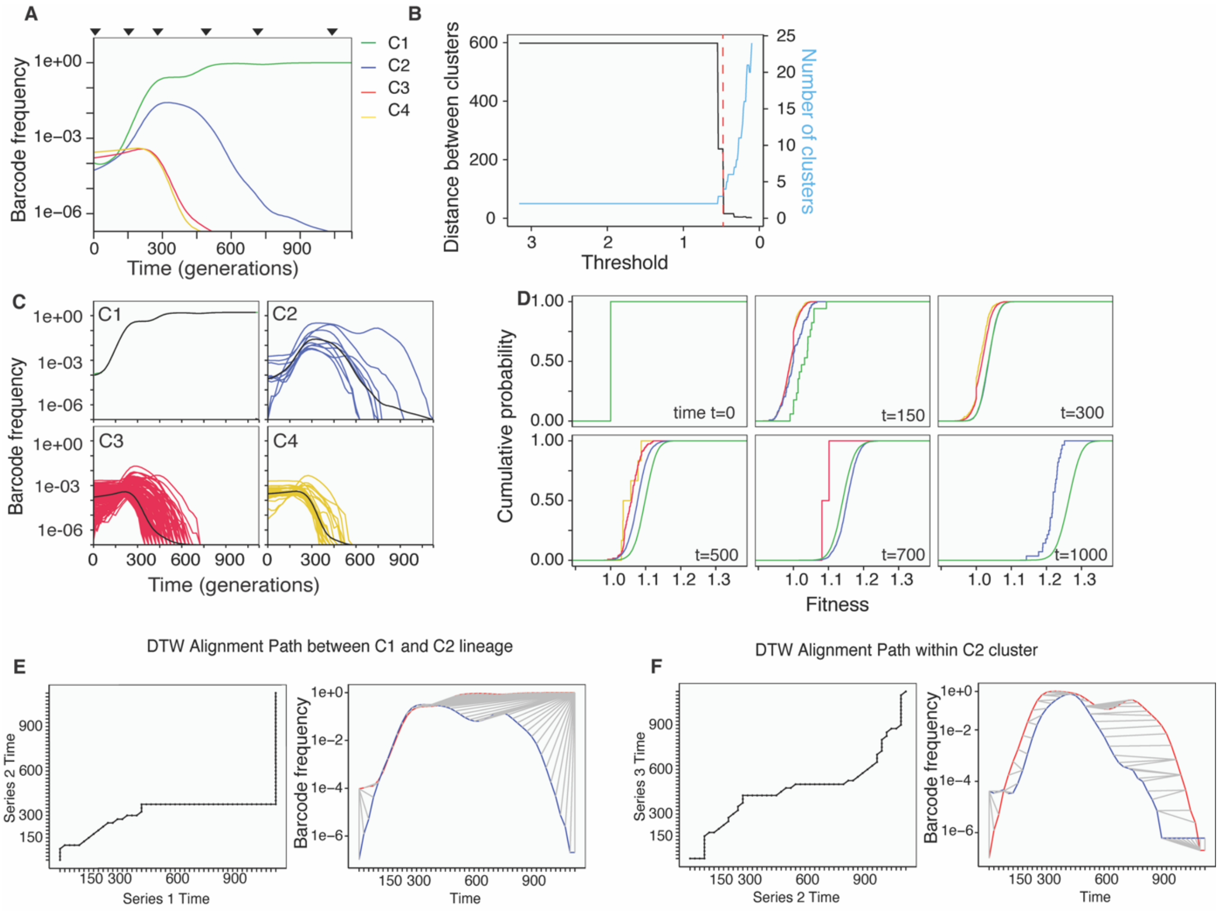

Supplementary Figure 6 Using Doblin to analyze high-resolution lineage data from evolutionary simulations with DTW distance

A) We re-analyzed the same high-resolution lineage data shown in Supplementary Figure 2, but instead of using Pearson correlation to compute pairwise distances, we applied Dynamic Time Warping (DTW) to construct the distance matrix. We then applied the full Doblin pipeline to identify clonal clusters and infer their consensus trajectories (centroids). This panel compares the centroids derived using DTW-based clustering to those obtained with Pearson-based clustering.**(B)** Hierarchical clustering quantification using the DTW-based distance matrix. The black curve shows the total Euclidean distance between cluster centroids, and the blue curve shows the number of clusters. Their intersection, marked by a red dashed line, indicates the optimal clustering cutoff, which yields four distinct clonal clusters (C1–C4). **(C)** Composition of each clonal cluster (C1–C4) as identified using the DTW-based distance matrix. **(D)** Cumulative distribution functions (CDFs) of cell fitness values within each clonal cluster at the specified time point, illustrating fitness variability across clusters. **E)** Examination of DTW alignment paths for the top two highest-frequency lineages. The first panel shows a C1 lineage that increased in frequency and aligns closely with a trajectory in C2. The second panel displays the DTW alignment path overlaid on their respective frequency trajectories, highlighting regions of temporal similarity and divergence.**(F)** Same as (E), but for the two highest-ranked lineages within the C2 clonal cluster. Their DTW alignment paths are shown to illustrate the degree of temporal coordination within the cluster.

2.2. Application II: Using Doblin to extract dominant lineage behaviors from an experimental evolution o*f E. coli*

We applied *Doblin* to high-resolution lineage data from *in vitro* evolution of *E. coli* under the antibiotic Trimethoprim (TMP) (0.1 μg ml^{-1} TMP, replicate 2) with a population size of ~3x10^{7} cells (Jasinska, et al., 2020). Unlike simulated data, the frequency trajectories derived from experimental data exhibit more complex evolutionary dynamics (Supplementary Figure 7A, Supplementary Figure 9B). Since *Doblin*'s aim is to estimate dominant lineage behaviors, we restricted our analysis to include only the lineages with a mean frequency exceeding 10^{-4} and persisting for at least 12 consecutive time points out of the 16. These cut-offs can be modified by the user to explore the effects of experimental noise that could strongly affect the dynamics of low-frequency barcodes. In Supplementary Figure 7B, we show the composition of the resulting clonal cluster, indeed revealing clusters that group barcodes with similar dynamics. The clonal lineage dynamics (Supplementary Figure 7A) are indicative of the presence of clonal interference. Indeed, certain lineages initially benefiting from positive selection (i.e. C4, C5 and C6) diminish later as higher-fitness competitors emerge (i.e. C1 and C2). Furthermore, the initial increase observed in C1, C4 and C6 may be caused by pre-existing beneficial mutations present at the start the experiment (standing genetic variation). After 100 generations, while the frequencies of C4 and C6 began to decline, C1 emerged as the dominant cluster. This observation suggests that mutations within C1 underwent selection, providing a competitive advantage to lineages within this cluster. In contrast, the frequencies of C2 and C3 increased later in the experiment, accompanied by significant fluctuations. This pattern indicates that lineages within these clonal clusters likely acquired *de novo* mutations which, unlike those in C1, failed to pass selection.

We also applied Doblin to compare the dominant clonal dynamics in two antibiotic concentrations, one at TMP = 0.1 μg ml^{-1} and another that has 1 order of magnitude lower (TMP = 0.01 μg ml^{-1}) from (Jasinska, et al., 2020). While genetic drift contributed to the loss of several individual low-frequency lineages in both conditions, it commenced later in the lower TMP condition (Supplementary Figure 8). The rate of barcode diversity loss is expected to correlate with the strength of selection pressure, which is the antibiotic concentration in this case. Consequently, the lower TMP concentration led to slower diversity collapse, leading to a longer period where clonal interference occurs. Supplementary Figure 8 demonstrates these expected behaviors, where the clonal cluster dynamics exhibit a slower sweep and more complex dynamics in the lower TMP concentration.

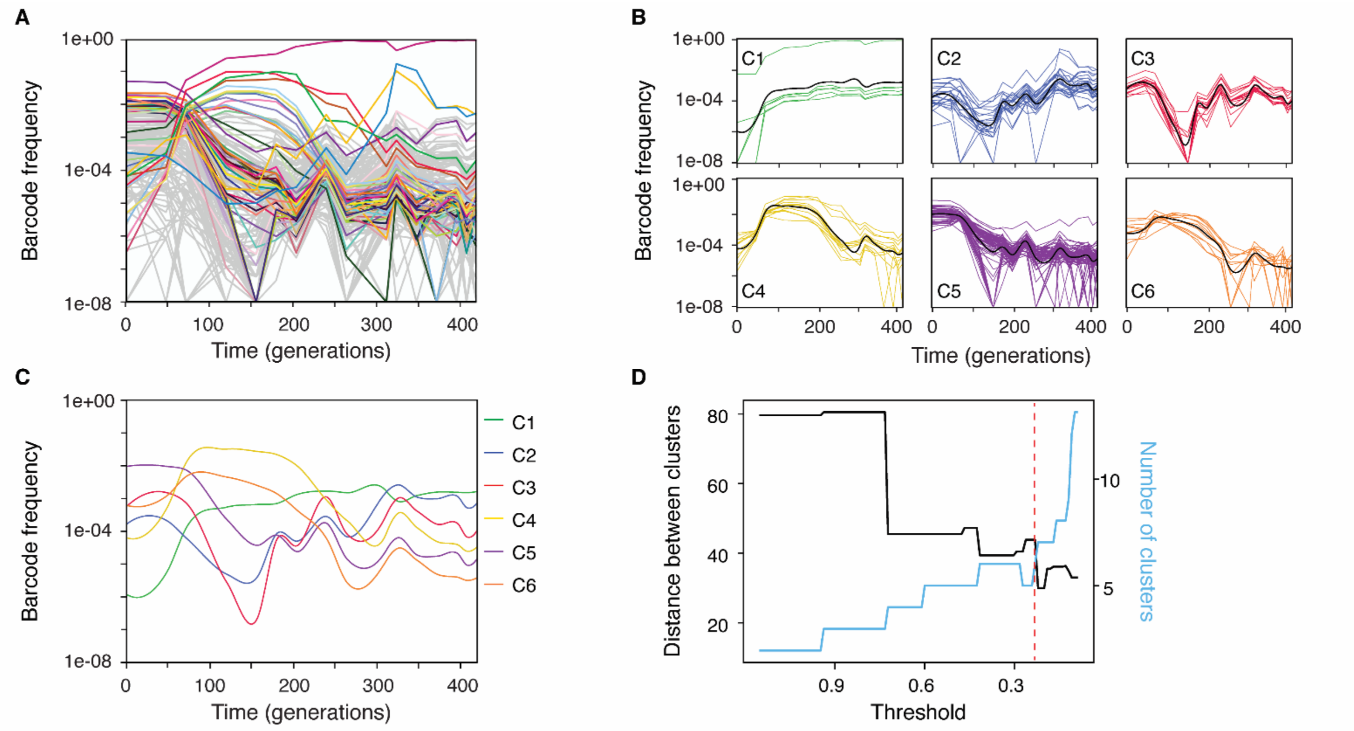

Supplementary Figure 7 Application II: Doblin applied to a month-long experimental evolution of *E. coli* under antibiotics.

(A) Frequency trajectories of barcoded lineages throughout a month-long evolution of *E. coli* under antibiotic (Trimethoprim = 0.1 μg ml-1 , replicate 2 in (Jasinska, et al., 2020)). The top 50 barcodes with a mean frequency over time greater than 10-4 are uniquely colored, whereas the rest are shown in gray. **(B)** Composition of the clonal clusters identified by Doblin. These clusters were obtained using a pairwise distance matrix based on Pearson's correlation. Only the lineages with mean frequency over time greater than 10-4 and persisting for at least 12 out of 16 time points were retained. Colored lines correspond to unique chromosomal barcodes in the cluster, while each cluster's black line denotes its LOESS average. The clonal clusters are ordered based on their average frequency at the final time point. Here, cluster 1 (C1) contains the barcode that manifested the clonal sweep. **(C)** averages, or consensus trajectories, of the 6 clonal clusters identified by Doblin. They reflect the dominant behaviors observed in the dataset. **(D)** The threshold (red dashed line) indicates the heuristically optimal clustering, resulting in 6 clusters (i.e. C1 – C6).

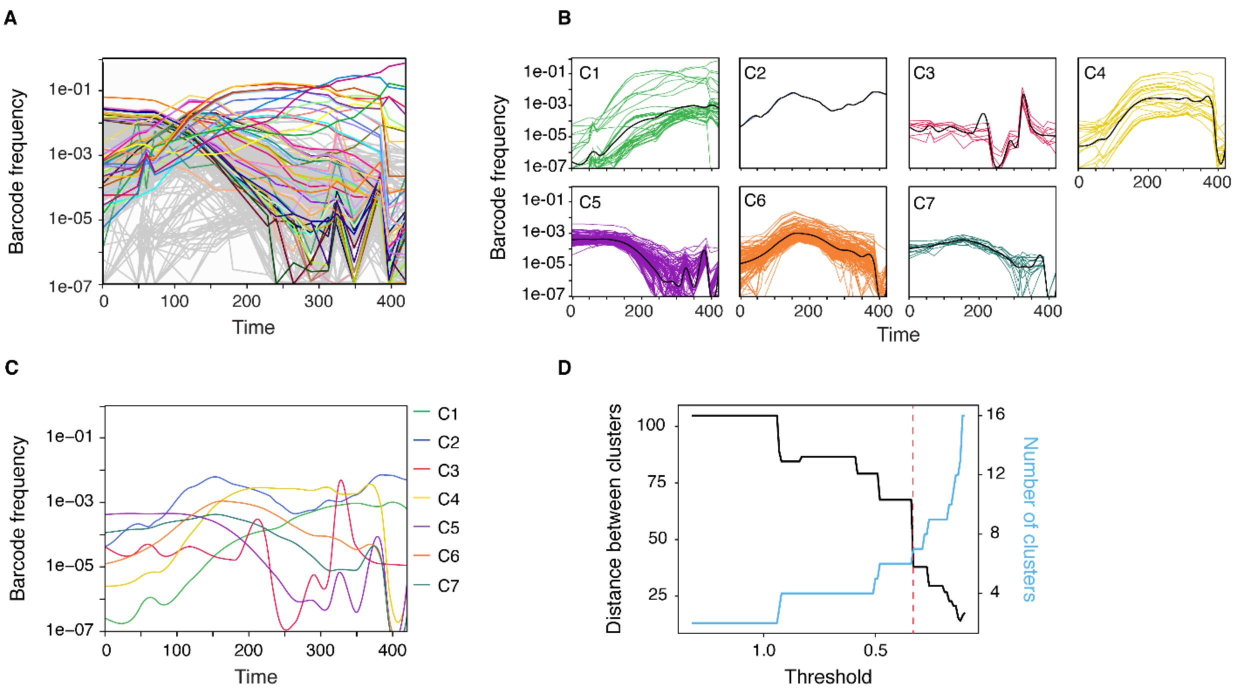

Supplementary Figure 8 Doblin applied to a month-long experimental evolution of *E. coli* under lower antibiotic regime.

(**A**) Similar to Supplementary Figure 7, but under 0.01 μg ml^{-1} of Trimethoprim (replicate 3) (Jasinska, et al., 2020). (**B**) Composition of the clonal clusters identified by *Doblin*. Only the lineages with mean frequency over time greater than 10^{-4} and persisting for at least 12 out of 17 time points were retained for the analysis. (**C**) LOESS averages of the 7 clonal clusters identified by *Doblin*. (**D**) Threshold (red dashed line) for optimal clustering, resulting in 7 clusters (i.e. C1 – C7).

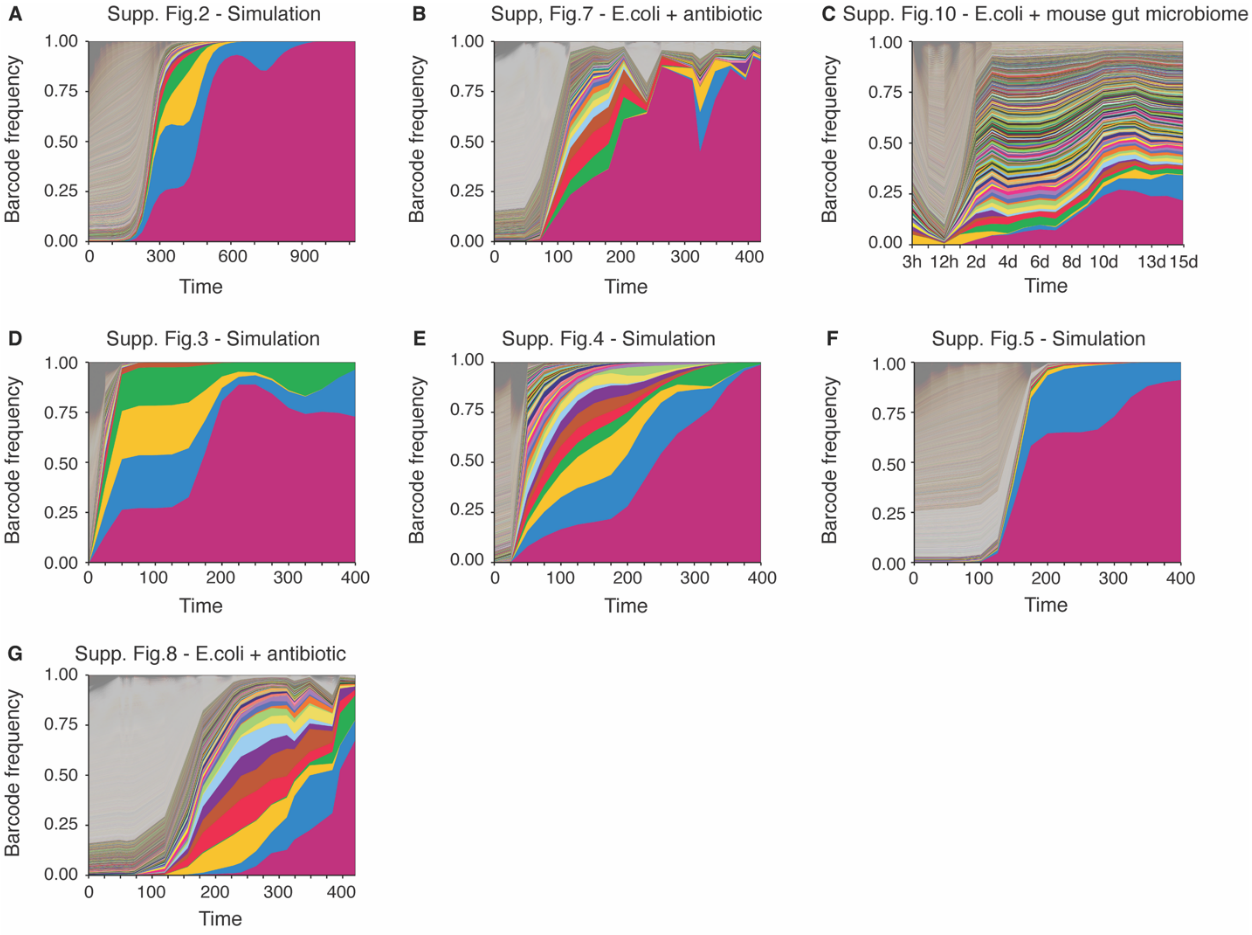

Supplementary Figure 9 Dynamics of barcoded lineages frequencies in simulation and experiments to which Doblin has been applied.

Each panel illustrates frequency trajectories represented as Muller plots, showcasing changes in frequencies over time on a linear scale. The colors used in these plots maintain consistency between the logarithmic and linear scale representations. Panel (**A**) displays the Muller plot of frequency trajectories in Supplementary Figures 2A, while Panels (**B**) through (**G**) represent the frequency trajectories of Supplementary Figures 7, Supplementary Figures 10, and Supplementary Figures 3 through 8, respectively.

2.3. Application III: Using *Doblin* to extract dominant behaviors from abundance time series of *E. coli* invading the gut microbiome of mouse

We applied Doblin to high-resolution lineage data obtained from a two-week *E. coli* evolution experiment in the mouse gut to examine how increased population heterogeneity influences population dynamics (Gencel, et al., 2025) (Supplementary Figure 10). Clonal clusters were identified using Pearson correlation with UPGMA hierarchical clustering. A notable observation is that clones which arrive early in the gut are not necessarily the eventual "winners" (i.e., the dominant clones) by the end of the experiment (Supplementary Figure 9C). Examination of the clonal clusters reveals that the first barcodes to colonize the gut are either eliminated from the population or persist at lower frequencies (Supplementary Figures 10B and 10C). This highlights the influence of transient colonization dynamics in shaping long-term outcomes. The dominant clonal cluster, C1, shows better adaptation to the gut environment. In contrast, clonal clusters C4

through C8 persist at lower frequencies and display substantial fluctuations. These variations may reflect ecological factors, such as interactions with other microbial species, or the effects of *de novo* mutations that arise during colonization.

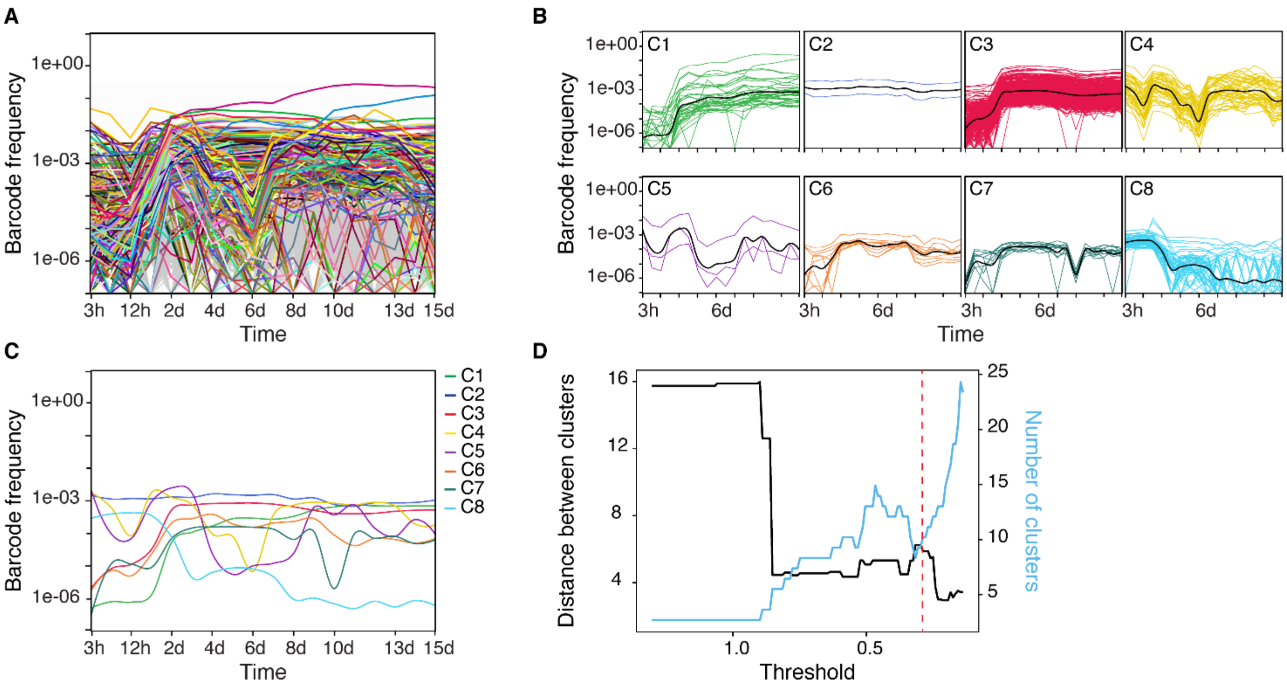

Supplementary Figure 10 Application III: Using Doblin to extract dominant behaviors from abundance time series of *E. coli* invading a mouse gut microbiome.

(A) Frequency of ~10^8 chromosomal barcoded cells of *E. coli* over 2-week period in mice gut with pre-existing microbiome (from rm1 of cohort 2) (Gencel, et al., 2025). The top 1000 barcodes with a mean frequency over time greater than $5x10^{-4}$ are colored uniquely, whereas the rest are shown in gray. **(B)** Composition of the clonal clusters identified by Doblin using a pairwise distance matrix based on Pearson's correlation. We retained only the lineages with mean frequency over time greater than $5x10^{-5}$ and persistent for at least 10 out of 18 time points. **(C)** The LOESS averages of the 8 identified clonal clusters correspond to the dominant behaviors of the dataset. **(D)** The threshold (red dashed line) corresponds to the heuristically optimal clustering, which generated 8 clusters (i.e. C1 – C8).

References

Dunn, J.C. Well-Separated Clusters and Optimal Fuzzy Partitions. *Cybernetics and Systems* 2008;4:95-104.

Gauthier, L., Di Franco, R. and Serohijos, A.W.R. SodaPop: a forward simulation suite for the evolutionary dynamics of asexual populations on protein fitness landscapes. *Bioinformatics* 2019;35(20):4053-4062.

Gencel, M.*, et al.* Quantifying the intra- and inter-species community interactions in microbiomes by dynamic covariance mapping. *Nature Communications (pre-print)* 2025.

Jasinska, W.*, et al.* Chromosomal barcoding of E. coli populations reveals lineage diversity dynamics at high resolution. *Nat Ecol Evol* 2020;4(3):437-+.

Kinsler, G.*, et al.* Extreme Sensitivity of Fitness to Environmental Conditions: Lessons from #1BigBatch. *J Mol Evol* 2023;91(3):293-310.

R Core Team, R. R: A language and environment for statistical computing. 2013.

Raihan, N. Determining the Optimal Number of Clusters for Time Series Datasets with Symbolic Pattern Forest. 2023.
